# Matrix-Dependent Sensitivity of Two Pan-Trematode PCR Assays for Detecting *Schistosoma* spp. in Clinical Human Samples

**DOI:** 10.3390/idr18020027

**Published:** 2026-03-27

**Authors:** Hagen Frickmann, Andreas Hahn, Kirsten Alexandra Eberhardt, Ulrike Loderstädt, Norbert Georg Schwarz, Ralf Matthias Hagen

**Affiliations:** 1 Department of Microbiology and Hospital Hygiene, Bundeswehr Hospital Hamburg, 22049 Hamburg, Germany; 2 Institute for Medical Microbiology, Virology and Hygiene, University Medicine Rostock, 18057 Rostock, Germany; andreas.hahn@uni-rostock.de; 3 Department of Gastroenterology, Hepatology and Infectious Diseases, University Hospital and Medical Faculty of the Heinrich Heine University, 40225 Düsseldorf, Germany; 4 Department of Tropical Medicine, Bernhard Nocht Institute for Tropical Medicine, 20359 Hamburg, Germany; 5 Institute of Infection Control and Infectious Diseases, University Medical Center Göttingen, 37075 Göttingen, Germany; ulrike.loderstaedt@med.uni-goettingen.de; 6 Independent Researcher, 67459 Böhl-Iggelheim, Germany; schwarznorbert@web.de; 7 Department of Microbiology and Hospital Hygiene, Bundeswehr Central Hospital Koblenz, 56070 Koblenz, Germany; ralfmatthiashagen@bundeswehr.org

**Keywords:** pan-trematode PCR, *Schistosoma mansoni* complex, *Schistosoma haematobium* complex, diagnostic accuracy, schistosomiasis, test comparison

## Abstract

Background: *Schistosoma* spp. are trematodes occurring in tropical endemic areas but can be imported to non-endemic regions as causes of travel-associated infections. In this study, two pan-trematode-specific real-time PCR assays were evaluated for their diagnostic sensitivity in detecting *Schistosoma* spp. DNA in diagnostic human samples. Methods: Two previously described pan-trematode-specific real-time PCR assays were comparatively assessed using diagnostic samples containing DNA of either the *S. haematobium* complex or the *S. mansoni* complex, as confirmed by *Schistosoma* species complex-specific real-time PCR. Results: Out of a total of 655 samples containing *Schistosoma* spp. DNA, positive signals in at least one of the two pan-trematode real-time PCR assays were recorded for 17 (2.6%) nucleic acid extractions. Although sensitivity was in the >90% range for stool samples, only a few individual blood plasma and serum samples, and none of the *Schistosoma* spp. DNA-containing tissue or urine samples, tested positive by pan-trematode PCR. The lower sensitivity of pan-trematode PCR compared with *Schistosoma* spp.-specific PCR was semi-quantitatively confirmed by higher cycle threshold (Ct) values in the former. When comparing samples with concordant versus discordant positive results for *Schistosoma* spp.-specific and pan-trematode PCR, Ct values of the *Schistosoma* spp.-specific PCR were lower in concordantly positive samples than in discordantly positive samples. Conclusions: While the assessed pan-trematode PCR assays showed insufficient sensitivity as screening tools for blood plasma, blood serum, tissue, and urine samples from individuals with suspected schistosomiasis, they were sufficiently sensitive when applied to stool samples, in which substantial amounts of target DNA, as indicated by low Ct values in the *Schistosoma* species complex-specific real-time PCR assays, can be expected. For screening for *Schistosoma* spp. DNA in sample materials other than stool, the use of highly sensitive target-specific PCR remains necessary.

## 1. Introduction

Human trematode infections, which occur focally in predominantly tropical endemic settings, represent a considerable diagnostic challenge. More than 100 trematode species from this helminth group have been reported to infect human individuals, involving various body compartments such as the blood, lungs, liver, and intestine [1,2,3]. In wildlife, several thousand different trematode species have been identified to date [4]. In human disease, the qualitatively and quantitatively most relevant trematode pathogens include *Fasciola* spp., which cause hepatic invasion [5], *Paragonimus* spp., which cause pulmonary invasion [6], and *Schistosoma* spp., which persist in human blood vessels [7,8,9,10].

Next to this considerable diversity, the morphological similarity of various trematode eggs, the restricted sensitivity of microscopic methods, and the partly ectopic location of helminth eggs make traditional parasitological diagnostic approaches poorly reliable [1,2], particularly for investigators with limited experience in non-endemic regions. Molecular diagnostic approaches have therefore become less investigator- dependent alternatives for many otherwise difficult-to-diagnose pathogens in diagnostic laboratories.

When the target organism is unknown and non-specific symptoms are compatible with a wide range of differential diagnoses, broad-spectrum diagnostic methods are desirable. Although pan-microbial PCR (polymerase chain reaction) with subsequent Sanger sequencing is well established for bacterial and fungal pathogens in microbiological diagnostic laboratories [11], this approach is used much less frequently for microbial parasites.

For parasitic helminths, efforts have been made to establish pan-helminthic PCRs, but these have been hampered by considerable sequence variability. In an attempt to cover at least the most abundant species relevant to human disease, group-specific real-time PCR assays for pathogenic nematodes, trematodes, and cestodes have recently been described and evaluated [12,13,14]. Such approaches typically target ribosomal sequences and the helminths’ cyclooxygenase (*cox*) gene. However, because human samples positive for helminths are rarely available, most in vitro evaluations have been limited to small sample sizes [12,13,14].

For several specific helminth species, molecular diagnostic approaches are much better established. The causative agents of human schistosomiasis are no exception. For the *S. mansoni* complex and *S. haematobium* complex [15], which cause the African forms of the disease, highly sensitive and specific real-time PCR assays targeting highly repetitive genomic sequences, even in serum, have been described [15,16,17]. These assays detect freely circulating helminth DNA in human serum samples. For the Eastern and South-Eastern Asian form caused by species of the *S. japonicum* complex [15], attempts to establish similarly sensitive assays have yielded fewer convincing results [18]. For the molecular diagnosis of *Schistosoma* spp. from stool or urine, where higher DNA concentrations can be expected, PCRs targeting less repetitive genetic elements such as ribosomal sequences are usually considered sufficiently sensitive [19,20,21], although such approaches may fail when applied to serum samples [21].

Reliable diagnosis of schistosomiasis is desirable for several reasons. The inflammatory immune response directed against eggs trapped in tissues, with granuloma formation, is central to the pathology of the disease and should be addressed early with therapy to prevent complications [7]. Whereas *S. mansoni* is primarily associated with intestinal schistosomiasis, *S. haematobium* is the major causative agent of urogenital disease [22]. According to the World Health Organization (WHO) [23], schistosomiasis transmission occurs in 79 countries, and more than 90% of affected individuals live in Africa. An estimated number of more than 250 million people worldwide are considered to require preventive chemotherapy. Praziquantel is the treatment of choice, and artemisinin derivatives used in combination have shown promising results as well [24]. Even when the disease is not completely cured, treatment exerts beneficial effects on its progression [25].

In terms of both frequency and disease severity, *Schistosoma* spp. is considered the second most relevant blood parasite after *Plasmodium* spp. in human patients [26]. Prolonged infections can lead to several medical consequences, including long-term immune modulation to carcinogenesis associated with the immune response to *S. haematobium*, while symptoms may be non-specific or even absent [27,28,29].

In the present study, we assessed the sensitivity of previously described pan-trematode real-time PCR protocols [13,14] when applied to diagnostic samples that were positive for *Schistosoma* spp.-specific DNA by highly sensitive target-specific real-time PCR [15,16,17]. By doing so, we aimed to evaluate the potential suitability of pan-trematode-specific PCR for screening purposes, including the detection of human schistosomiasis.

## 2. Materials and Methods

### 2.1. Study Design and Sample Materials

The study was designed as a head-to-head test comparison. The sample materials comprised residual DNA eluates that had previously tested positive for *Schistosoma* spp.-specific sequences by real-time PCR targeting either the *Dra1* sequence of the *S. haematobium* complex or the *Sm1-7* sequence of the *S. mansoni* complex [17] in the diagnostic laboratory of the Bundeswehr Hospital Hamburg. Nucleic acid extraction had been performed using the EZ1 Virus Mini Kit v2.0 Kit (Qiagen, Hilden, Germany) on an automated EZ1 Advanced system (Qiagen, Hilden, Germany) for blood plasma, blood serum and urine samples; the EZ1&2 DNA tissue kit protocol on EZ1 automates (Qiagen, Hilden, Germany) after bead-beating-based tissue lysis for tissue samples; and the QiaAMP DNA Stool Mini Kit (Qiagen, Hilden, Germany) for stool samples, each according to the manufacturer’s instructions. Samples were included if sufficient residual DNA was available for the diagnostic approach described below. There were no exclusion criteria. In line with the ethical clearance detailed below, no patient-specific data were provided, which represents an acknowledged deviation from the STARD criteria for diagnostic studies [30]. The vast majority of samples were obtained from individuals from sub-Saharan Africa (blood plasma from Madagascans, blood serum and tissue samples from Ghanaians and individuals from Côte d’Ivoire), whereas the proportion of samples from travelers and migrants was quantitatively negligible and comprised 47 serum samples, 3 plasma samples, as well as all 2 urine samples and all 11 stool samples. Only the type of sample (e.g., stool, blood serum, blood plasma) was systematically recorded.

Consistent with this design, there were no specific patient-related inclusion criteria in terms of clinical presentation, disease status, or geographic origin or travel history for this laboratory-based assessment. The abundance of *Schistosoma* spp.-specific DNA within the sample, as determined by target-specific real-time PCR, was the sole inclusion criterion. Systematic testing for the DNA of other helminths in general, and other trematodes in particular, was not performed; therefore, we refrained from assessing the specificity of the investigated pan-trematode PCRs.

### 2.2. Laboratory Diagnostics

All DNA eluates were stored at −80 °C until they were tested with real-time PCR for the *Dra1* sequence of the *S. haematobium* complex and the *Sm1-7* sequence of the *S. mansoni* complex [17] to confirm the presence of *Schistosoma* spp.-specific DNA in the residual samples. Samples were included in further analyses if at least one positive real-time PCR signal was obtained. In such cases, two pan-trematode-specific real-time PCRs were performed as described previously [13,14]. Details of all real-time PCR assays applied are summarized in Appendix A, Table A1 and Table A2. Real-time PCR results were evaluated both qualitatively and semi-quantitatively using cycle threshold (Ct) values. Ct values were considered likely target-specific if they were associated with a sigmoid-shaped amplification curve, without the use of a predefined cut-off.

All PCRs were run on Corbett Q real-time PCR devices, and each run included a plasmid-based positive control (sequence inserts in a pEX A128 vector backbone; Eurofins Genomics, Luxembourg; details on the sequence inserts in Appendix A, Table A1) and a PCR-grade water-based negative control for quality control purposes. A previously described *Phocid herpesvirus* DNA-targeting real-time PCR [31] was added as an inhibition control assay, detecting the respective target DNA spiked into the diagnostic samples.

Similar to routine diagnostic conditions, each sample was analyzed in a single run without duplicate testing.

### 2.3. Statistics

The sensitivity estimates of both pan-trematode PCRs for *Schistosoma* spp. DNA-containing sample materials were calculated. In addition, Ct values of *Schistosoma* spp.-specific PCR associated with positive versus negative pan-trematode PCR results were compared. Non-parametric tests were applied, including Mann–Whitney tests for unpaired samples, Wilcoxon matched-pairs tests for paired samples, and calculation of Spearman’s rho for correlation analysis. All calculations were performed using GraphPad InStat, version 3.06 (GraphPad Software, Inc., San Diego, CA, USA).

### 2.4. Ethics

All procedures were carried out in accordance with the Declaration of Helsinki and all its amendments. Ethical approval was granted by the Medical Association of Hamburg, Germany (reference number: WF-011/19, obtained on 11 March 2019), allowing anonymous use of residual sample materials for test comparison purposes without the requirement for informed consent.

## 3. Results

### 3.1. Sample Material Characterization

A total of 655 residual DNA samples were included in the study, all of which showed positive real-time PCR results in at least one of the *S. mansoni* complex-specific or *S. haematobium* complex-specific assays. The residual DNA had been extracted from 577 blood plasma samples, 88 blood serum samples, 51 placental tissue samples (hereafter referred to as “tissues”), 11 stool samples, and 2 urine samples. All blood plasma and stool samples were positive for the *S. mansoni* complex, whereas the 2 urine samples were positive for the *S. haematobium* complex. Positive results for both *Schistosoma* complexes were obtained in tissue and serum samples. Specifically, 50 tissue samples were positive for the *S. haematobium* complex and 1 tissue sample for the *S. mansoni* complex, while among serum samples, 62 were positive for the *S. mansoni* complex only, 18 for the *S. haematobium* complex only, and 8 for both the *S. mansoni* and the *S. haematobium* complex.

### 3.2. Sensitivity of the Applied Pan-Trematode PCR Assays

As detailed in Table 1, at least one out of the two pan-trematode PCR assays yielded a positive signal in 17 of 655 samples (2.6%). These 17 samples consisted of 11 stool samples, 4 blood plasma samples, and 2 blood serum samples. No positive pan-trematode PCR results were obtained from *Schistosoma* spp. DNA-containing tissue samples or from the urine samples. The individual PCR results from these 17 samples are shown in Table 1.

Pan-trematode assay 1 yielded 15 positive results, and pan-trematode assay 2 yielded 13 positive results, corresponding to overall sensitivities of 2.3% for assay 1 and 2.0% for assay 2 across all 655 *Schistosoma* spp. DNA-containing samples. When analysis was restricted to samples containing *S. mansoni*-specific DNA only, the sensitivities were 2.4% for pan-trematode PCR 1 and 2.3% for pan-trematode PCR 2. For samples containing *S. haematobium* complex DNA only, sensitivities of 1.4% for pan-trematode PCR 1 and 0% for pan-trematode PCR 2 were observed. These data are summarized in Table 2. Details on concordant and discordant real-time PCR results are provided in Appendix A, Table A3.

A more differentiated pattern emerged when the various sample materials were considered separately, as shown in Table 3. For stool samples containing *S. mansoni* complex DNA alone, sensitivity was 100% for pan-trematode PCR 1 and 90.9% for pan-trematode PCR 2. For blood plasma samples containing *S. mansoni* complex DNA, sensitivities were 0.6% and 0.4% for pan-trematode PCR 1 and 2, respectively. For blood serum samples containing *S. mansoni* complex DNA and *S. haematobium* complex DNA, sensitivities of 0% and 3.8% for pan-trematode PCR 1 and 1.4% and 0% for pan-trematode PCR 2, respectively, were calculated. Sensitivity was 0% for both pan-trematode PCRs when applied to tissue samples containing either *S. mansoni* complex DNA or *S. haematobium* complex DNA, as well as to urine samples containing *S. haematobium* complex DNA. Stool or blood plasma samples containing *S. haematobium* complex DNA and urine samples containing *S. mansoni* complex DNA were not available, and sensitivities for these subgroups could therefore not be determined.

### 3.3. Characterization of Matching and Mismatching PCR Results by Cycle Threshold Value Comparison

As shown in Table 4, a correlation between Ct values of the *Schistosoma* spp.-specific PCRs and those of pan-trematode PCRs was observed. Statistical significance was confirmed for both pan-trematode assays in the analyses of all stool samples containing *S. mansoni* complex DNA as well as in the corresponding analyses of all samples containing *S. mansoni* complex DNA. The lack of significance for blood plasma samples containing *S. mansoni* complex DNA was attributable to the very low number of positive samples.

To obtain a crude estimation of sensitivity differences between the *Schistosoma* spp.-specific real-time PCRs and the pan-trematode PCRs, inter-assay Ct value differences were calculated, as presented in Appendix A, Table A4. For *S. mansoni* complex DNA-containing stool samples, the mean ± standard deviation (SD) Ct difference was 8.1 ± 1.4 cycles for pan-trematode PCR 1 and 10.3 ± 2.2 cycles for pan-trematode PCR 2, with a Wilcoxon matched-pairs test *p*-value of 0.0039. For *S. mansoni* complex DNA-containing blood plasma samples, the corresponding means and SDs were 4.7 ± 1.5 and 10 ± 0 cycles, respectively, but a *p*-value could not be estimated. When comparing the Ct value differences for pan-trematode PCR 1 between stool and blood plasma samples, the Mann–Whitney test yielded a *p*-value of 0.0239, whereas a *p*-value could not be estimated for pan-trematode PCR 2. As shown in Appendix A, Table A5, comparison of Ct values between concordantly and discordantly positive pan-trematode PCR results indicated that discrepant results tended to occur at high Ct values, whereas good concordance was generally observed for Ct values of 30 or lower.

As indicated in Table 3, a sample-stratified comparison of *Schistosoma* spp.-specific Ct values in cases of concordant versus discordant pan-trematode PCR results yielded a statistically significant difference only for the *S. mansoni* complex PCR in combination with pan-trematode PCR 1 when applied to blood plasma samples. For most other analyses, the low number of positive results precluded formal statistical testing, although a trend towards lower Ct values in *Schistosoma*-specific PCR in cases with concordant pan-trematode PCR results was evident. In a crude comparison of *S. mansoni* complex PCR Ct values between samples with concordant and discordant pan-trematode PCR results, the Mann–Whitney test yielded a *p*-value of <0.0001, with mean (±SD) Ct values of 24.3 (±6.3) for concordant samples and 35.9 (±3.1) for discordant samples.

## 4. Discussion

The study was conducted to evaluate the sensitivity of two published pan-trematode PCR assays when applied to samples containing *Schistosoma* spp. DNA, as indicated by highly sensitive *Schistosoma* spp.-specific real-time PCR [15,16,17]. The investigation yielded several key findings.

First, the results confirmed the expected high-sensitivity of the *Schistosoma* species complex-specific assays [15,16,17] compared with the pan-trematode assays. As outlined in the introduction, the *Schistosoma* species complex-specific assays targeted gene regions present in thousands of copies per parasite [15,16,17], which largely accounts for their increased sensitivity relative to the pan-trematode assays targeting ribosomal genes that occur at much lower copy numbers. Good concordance between *Schistosoma* spp. PCR and pan-trematode PCR was observed only in stool samples, in which substantial amounts of targeted DNA corresponded to low Ct values in *Schistosoma* spp.-specific PCR. In stool samples, the sensitivity of the pan-trematode assays was therefore considered sufficient for screening purposes, although the small number of available *Schistosoma*-positive stool samples limits the interpretability of these results. For all other sample matrices—blood plasma, blood serum, tissues, and urine—this was not the case.

Interestingly, the recorded sensitivity of pan-trematode PCR was slightly higher in blood serum than in blood plasma. However, the low total number of positive pan-trematode PCR results in either sample type complicates interpretation. Given the strong overall association of positive pan-trematode PCR results with low Ct values in *Schistosoma* spp.-specific PCR, the observation may simply reflect the incidental occurrence of higher *Schistosoma* spp. target DNA amounts in the serum samples assessed.

Second, when the two pan-trematode assays [13,14] were compared for their diagnostic sensitivity in *Schistosoma* spp. DNA-containing human samples, these assays yielded largely concordant results. Discordant results were associated with high Ct values, indicating low target DNA quantity close to the technical detection limit. Notably, *S. haematobium* complex DNA was detected by pan-trematode PCR 1 only, which targets the helminth’s 18S rRNA gene. As this was the only positive pan-trematode PCR signal in a *Schistosoma haematobium* complex DNA-containing sample, it remains unclear whether this reflects genuinely higher sensitivity or simply an incidental finding. Of note, when comparing Ct values across different real-time PCR assays, it must be kept in mind that Ct values may vary depending on multiple factors, including oligonucleotide match to the target, varying PCR efficiency, and differing target sequence copy numbers within the organism.

The study has several limitations. First, regarding the *Schistosoma* species complex-specific PCR as a “gold standard” with the hypothetical assumption of 100% specificity is an oversimplification, as also shown by previous validation studies [15,16,17]. However, given the relatively consistent results and the low number of concordantly positive samples in both *Schistosoma* spp.-specific PCR and pan-trematode PCR, the associated bias was considered acceptable, and modeling approaches for test comparisons without a true gold standard were not used. Second, the available samples showed a markedly uneven distribution of sample types, of *S. mansoni* complex versus *S. haematobium* complex detections, and of low versus high Ct values as semi-quantitative indicators of target DNA amounts. In particular, the low Ct value range of *Schistosoma* spp.-specific PCR, where concordant results with pan-trematode PCR are most likely, was represented by only a low number of samples. Third, and as already noted, ethical requirements for this study prevented the inclusion of patient-specific data, which constitutes a deviation from the STARD criteria for diagnostic studies [30] and complicates the interpretation of the findings. Fourth, as an inherent feature of the study design, the assessment focused exclusively on the sensitivity of the pan-trematode PCR assays, without a dedicated evaluation of specificity. *Schistosoma* spp.-specific PCR assays are not suitable as reference standards for specificity assessments of pan-trematode PCRs, because they do not exclude the presence of DNA from trematodes other than *Schistosoma* species. Fifth, due to the limited availability of residual sample materials, differential effects of various nucleic acid extraction strategies on factors affecting PCR sensitivity, such as matrix-associated PCR inhibition or target DNA degradation, could not be assessed. Despite this limitation, the Ct value analysis strongly suggests that it was primarily the relatively high target DNA amounts, as indicated by low Ct values, that accounted for the better performance of pan-trematode PCR in stool compared with other sample matrices. Sixth, although robust diagnostic accuracy has previously been demonstrated for the duplex real-time PCR assay used as the reference standard [17], it does not meet the strict definition of a true gold standard with 100% sensitivity and specificity. Verification bias should therefore be taken into account when interpreting the study results.

## 5. Conclusions

In spite of the limitations outlined above, the study clearly indicates that the diagnostic sensitivity of pan-trematode PCR is unacceptably low for screening purposes when applied to human sample materials other than stool from patients with schistosomiasis, compared with highly sensitive *Schistosoma* species complex-specific PCR assays. At least for *Schistosoma* mansoni complex DNA-containing samples, 18S rRNA gene-specific and 28S rRNA gene-specific pan-trematode PCRs show comparable sensitivity, with a tendency toward slightly more positive results when using the 18S rRNA gene-specific assay. When applying the described pan-trematode PCR assays with human stool samples, it should be noted that acceptable sensitivity was demonstrated only for *S. mansoni* complex DNA, and on the basis of a limited number of positive samples. Regarding other enteric trematode infections, future evaluations in endemic settings are still required.

## Figures and Tables

**Table 1:** Residual sample material and Ct values of recorded real-time PCR results of samples being concordantly positive in *Schistosoma* spp.-specific PCR and at least one out of the two pan-trematode PCRs.

Sample Number	Sample Material, from Which the Residual DNA Eluate Was Obtained	Ct Value Measured with Pan-Trematode PCR 1	Ct Value Measured with Pan-Trematode PCR 2	Ct Value Measured with *S. haematobium* Complex PCR	Ct Value Measured with *S. mansoni* Complex PCR
1	Stool	26	27	-	19
2	Stool	33	35	-	25
3	Stool	30	30	-	20
4	Stool	24	28	-	15
5	Stool	35	-	-	26
6	Stool	28	31	-	20
7	Stool	29	34	-	21
8	Stool	26	29	-	16
9	Stool	27	29	-	20
10	Stool	31	33	-	26
11	Stool	28	29	-	20
12	Blood plasma	37	-	-	32
13	Blood plasma	38	42	-	32
14	Blood plasma	38	-	-	35
15	Blood plasma	-	45	-	35
16	Blood serum	-	43	-	27
17	Blood serum	40	-	23	-

Ct = cycle threshold.

**Table 2:** Sensitivity of the two assessed pan-trematode-specific real-time screening PCR assays in samples containing *Schistosoma* spp. DNA.

Assay	Total Tested (*n*)	Positives (*n*)	Sensitivity in % (95% CI)
Pan-trematode PCR 1	655	15	2.3% (1.3%, 3.8%)
Pan-trematode PCR 2	655	13	2.0% (1.1%, 3.4%)
Pan-trematode PCR 1 (compared to *S. mansoni* complex alone)	577 *	14	2.4% (1.3%, 4.0%)
Pan-trematode PCR 2 (compared to *S. mansoni* complex alone)	577 *	13	2.3% (1.2%, 3.8%)
Pan-trematode PCR 1 (compared to *S. haematobium* complex alone)	70 *	1	1.4% (0%, 7.7%)
Pan-trematode PCR 2 (compared to *S. haematobium* complex alone)	70 *	0	0% (-)

*n* = number included. 0.95 CI = 95% confidence interval. * No addition to 655 due to 8 samples being positive for both *S. mansoni* complex and *S. haematobium* complex.

**Table 3:** Pan-trematode PCR results and cycle threshold (Ct) value comparisons stratified by sample material.

*Schistosoma* spp.-Specific PCR (Target Sequence)	Pan-Trematode-Specific PCR	Sample Material (*n*)	Number and Proportion of Pan-Trematode PCR Positives, *n*/*n* (%)	Ct Values of Pan-Trematode PCR Positives, Mean (±SD)	Ct Values of Pan-Trematode PCR Negatives, Mean (±SD)	*p*-Value
*S. mansoni* complex (*Sm1-7*)	PCR 1	Stool (11)	11/11 (100%)	20.7 (±3.7)	n.a.	n.e.
*S. mansoni* complex (*Sm1-7*)	PCR 2	Stool (11)	10/10 (90.9%)	20.2 (±3.4)	26 (±0)	n.e.
*S. mansoni* complex (*Sm1-7*)	PCR 1	Blood plasma (503)	3/503 (0.6%)	33.0 (±1.7)	36.4 (±2.8)	0.0371
*S. mansoni* complex (*Sm1-7*)	PCR 2	Blood plasma (503)	2/503 (0.4%)	33.5 (±2.1)	36.4 (±2.8)	0.1346
*S. mansoni* complex *(Sm1-7*)	PCR 1	Blood serum (70/88 *)	0/70 (0%)	n.a.	32.4 (±3.4)	n.e.
*S. mansoni* complex (*Sm1-7*)	PCR 2	Blood serum (70/88 *)	1/70 (1.4%)	27 (±0)	32.5 (±3.3)	n.e.
*S. haematobium* complex (*Dra1*)	PCR 1	Blood serum (26/88 *)	1/26 (3.8%)	23 (±0)	33.0 (±3.9)	n.e.
*S. haematobium* complex (*Dra1*)	PCR 2	Blood serum (26/88 *)	0/26 (0%)	n.a.	32.6 (±4.3)	n.e.
*S. mansoni* complex (*Sm1-7*)	PCR 1	Tissue (1)	0/1 (0%)	n.a.	37 (±0)	n.e.
*S. mansoni* complex (*Sm1-7*)	PCR 2	Tissue (1)	0/1 (0%)	n.a.	37 (±0)	n.e.
*S. haematobium* complex (*Dra1*)	PCR 1	Tissue (50)	0/50 (0%)	n.a.	31.2 (±1.6)	n.e.
*S. haematobium* complex (*Dra1*)	PCR 2	Tissue (50)	0/50 (0%)	n.a.	31.2 (±1.6)	n.e.
*S. haematobium* complex (*Dra1*)	PCR 1	Urine (2)	0/2 (0%)	n.a.	27.5 (±3.5)	n.e.
*S. haematobium* complex (*Dra1*)	PCR 2	Urine (2)	0/2 (0%)	n.a.	27.5 (±3.5)	n.e.

Ct = cycle threshold. *n* = number. *p*-value = significance level applying Mann–Whitney testing. SD = standard deviation. * Overlap of 8 blood serum samples showing positive real-time PCR results for both *S. mansoni* complex and *S. haematobium* complex. n.a. = not applicable. n.e. = not estimable.

**Table 4:** Correlation of cycle threshold (Ct) values of *Schistosoma* spp.-specific real-time PCRs and pan-trematode real-time PCRs.

	Sample Material	Number of Points	Spearman r, Corrected for Ties (95% Confidence Interval)	*p*-Value
*S. mansoni* complex PCR and pan-trematode PCR 1	Stool	11	0.9370 (0.7612, 0.9845)	<0.0001
*S. mansoni* complex PCR and pan-trematode PCR 2	Stool	10	0.8609 (n.e.)	0.0022
*S. mansoni* complex PCR and pan-trematode PCR 1	Blood plasma	3	0.5000 (n.e.)	>0.9999
*S. mansoni* complex PCR and pan-trematode PCR 1	All	14	0.9665 (0.8913, 0.9900)	<0.0001
*S. mansoni* complex PCR and pan-trematode PCR 2	All	13	0.9328 (0.7784, 0.9808)	<0.0001

*p*-value = significance as calculated for the Spearman r assessments. n.e. = not estimable.

## Data Availability

All relevant data are provided in the manuscript or its tables in Appendix A. Raw data can be made available at a reasonable request.

## Abbreviations

The following abbreviations are used in this manuscript:

0.95 CI	95% confidence interval
Ct	Cycle threshold
HIV	Human immunodeficiency virus
max.	Maximum
min.	Minute/minimum
n.a.	Not applicable
n.e.	Not estimable
*p*-value	Significance level
PCR	Polymerase chain reaction
ref.	Reference
SD	Standard deviation
sec.	Second
spp.	Species (plural)
WHO	World Health Organization

## Appendix A

**Table A1:** Target genes, calculated detection limits, and oligonucleotides used for the applied real-time PCR assays for trematodes and for *Schistosoma* spp. Hyphens in the oligonucleotide sequences have been inserted to increase the readability, not to delineate codon triplets.

PCR Target	Pan-Trematode PCR 1
Target gene	18S rRNA gene
Detection limit	Not applicable due to species-dependent variability
Forward primer	5′-WGA-GGC-TCC-GTA-ATT-CGA-3′
Reverse primer 1	5′-TGC-GAY-CGC-ACK-ACC-C-3′
Reverse primer 2	5′-CRY-AGC-CAT-SCG-ACC-C-3′
Probe and modifications	5′-FAM-YCA-ACT-ACG-AGC-TTT-TKA-ACT-GCA-RCA-ACT-BHQ1-3′
Positive control plasmid insert	5′-AAT-ACG-GAT-ACG-GGA-CTC-ACT-AGA-GGC-TCC-GTA-ATT-CGA-ATG-AGT-ACA-ATT-TAA-ATC-CTT-TAA-CGA-GGA-TCA-ACT-GGA-GGG-CAA-GTC-TGG-TGC-CAG-CAG-CCG-CGG-TAA-CTC-CAG-CTC-CAG-AAG-CGT-ATA-TTA-AAG-TTG-TTG-CAG-TTC-AAA-AGC-TCG-TAG-TTG-GAT-CTG-GGT-CGC-ATG-GCT-ACA-TGC-CGT-CGC-TCG-TGG-GTC-TGG-CCT-GGT-TAC-3′
GenBank accession number used for the insert	AJ004969.1
Reference	[14]
PCR target	Pan-trematode PCR 2
Target gene	28S rRNA gene
Detection limit	not applicable due to species-dependent variability
Forward primer	5′-AGG-CAA-TGT-GGT-GTT-YAG-GT-3′
Reverse primer	5′-CAC-AAA-CAA-CCC-GAC-TCC-AA-3′
Probe and modifications	5′-FAM-TGG-CCC-AND-GAG-GGT-GAA-AGG-C-BHQ1-3′
Positive control plasmid insert	5′-ATT-GGT-CAC-TAG-GCA-ATG-TGG-TGT-TCA-GGT-CGT-TCC-GCG-GAG-GTG-CTG-CTC-CAT-TCC-AAG-TCC-AGC-AAT-GAG-TAC-GGT-AAT-GCT-GAC-ATG-GCC-CAA-AGA-GGG-TGA-AAG-GCC-CGT-TGG-GGT-GGA-GAG-GCA-GAA-ATG-ACA-GCA-CCT-TCC-TGG-ATA-GAC-CTT-GGA-GTC-GGG-TTG-TTT-GTG-AAT-GCA-GCC-CAA-3′
GenBank accession number used for the insert	MK450524.1
Reference	[13]
PCR target	*S. mansoni* complex PCR
Target gene	*Sm1-7* sequence
Detection limit	5 × 10^2^/µL
Forward primer	5′-CCA-CGC-TCT-CGC-AAA-TAA-TCT-3′
Reverse primer	5′-CAA-CCG-TTC-TAT-GAA-AAT-CGT-TGT-3′
Probe and modifications	5′-FAM-TCC-GAA-ACC-ACT-GGA-CGG-ATT-TTT-ATG-AT-BHQ1-3′
Positive control plasmid insert	5′-TCC-GAC-CAA-CCG-TTC-TAT-GAA-AAT-CGT-TGT-ATC-TCC-GAA-ACC-ACT-GGA-CGG-ATT-TTT-ATG-ATG-TTT-GTT-TTA-GAT-TAT-TTG-CGA-GAG-CGT-GGG-CGT-TA-3′
GenBank accession number used for the insert	M61098.1
Reference	[16]
PCR target	*S. haematobium* complex PCR)
Target gene	*Dra1* sequence
Detection limit	5 × 10^2^/µL
Forward primer	5′-GAT-CTC-ACC-TAT-CAG-ACG-AAA-C-3′
Reverse primer	5′-TCA-CAA-CGA-TAC-GAC-CAA-C-3′
Probe and modifications	5′-JOE-TGT-TGG-TGG-AAG-TGC-CTG-TTT-CGC-AA-BHQ1-3′
Positive control plasmid insert	5′-AAATTGGATCTCACCTATCAGACGAAACAAAGAAAATTTTAAAATTGTTGGTGGAAGTGCCTGTTTCGCAATATCTCCGGAATGGTTGGTCGTATCGTTGTGAAAATTG-3′
GenBank accession number used for the insert	DQ157698.1
Reference	[15]

**Table A2:** Reaction mixes and run conditions for the real-time screening PCR assays for trematodes and *Schistosoma* spp.

	Pan-Trematode PCR 1	Pan-Trematode PCR 2	*Schistosoma mansoni* Complex and *S. haematobium* Complex PCR
Reaction chemistry			
Master Mix	HotStar master mix (Qiagen, Hilden, Germany)	HotStar master mix (Qiagen, Hilden, Germany)	HotStar master mix (Qiagen, Hilden, Germany)
Reaction volume (µL)	20	20	20
Forward primer concentration (mol)	2.5 × 10^−7^	5 × 10^−7^	5 × 10^−7^
Reverse primer concentration (mol)	2.5 × 10^−7^	5 × 10^−7^	5 × 10^−7^
Probe concentration (mol)	6 × 10^−6^	2 × 10^−7^	3 × 10^−7^
Final Mg^2+^ concentration (mM)	5.0	5.0	6.0
Bovine serum albumin (g/L)	-	-	0.005
Eluate volume (µL)	2.0	4.0	5.0
Run conditions			
Initial denaturation	95 °C for 15 min	95 °C for 15 min	95 °C for 15 min
Cycle numbers	45	55	40
Denaturation	95 °C for 15 s	95 °C for 45 s	95 °C for 15 s
Annealing	60 °C for 30 s	55 °C for 45 s	60 °C for 60 s
Amplification	72 °C for 30 s	Combined with annealing	72 °C for 60 s
Hold	40 °C for 20 s	40 °C for 20 s	40 °C for 30 s

min = minute, s = second.

**Table A3:** Cross-table detailing mismatches between real-time pan-trematode PCRs. Green = matching results. Red = mismatching results.

All Samples			
		Pan-trematode PCR 1	
		Negative	Positive
Pan-trematode PCR 2	Negative	638	4
	Positive	2	11
Samples positive for the *S. mansoni* complex only			
		Pan-trematode PCR 1	
		Negative	Positive
Pan-trematode PCR 2		561	3
		2	11
Samples positive for the *S. haematobium* complex only			
		Pan-trematode PCR 1	
		Negative	Positive
Pan-trematode PCR 2	Negative	69	1
	Positive	0	0
Samples positive for both the *S. mansoni* complex and *S. haematobium* complex			
		Pan-trematode PCR 1	
		Negative	Positive
Pan-trematode PCR 2	Negative	8	0
	Positive	0	0

**Table A4:** Differences in the recorded Ct values of positive *Schistosoma* spp.-specific and pan-trematode real-time PCR results of samples testing concordantly positive.

Sample Number	Sample Material, from Which the Residual DNA Eluate Was Obtained	Ct Value Difference in Pan-Trematode PCR 1 and *S. haematobium* Complex PCR	Ct Value Difference in Pan-Trematode PCR 1 and *S. mansoni* Complex PCR	Ct Value Difference in Pan-Trematode PCR 2 and *S. haematobium* Complex PCR	Ct Value Difference in Pan-Trematode PCR 2 and *S. mansoni* Complex PCR
1	Stool	-	7	-	8
2	Stool	-	8	-	10
3	Stool	-	10	-	10
4	Stool	-	9	-	13
5	Stool	-	9	-	-
6	Stool	-	8	-	11
7	Stool	-	8	-	13
8	Stool	-	10	-	13
9	Stool	-	7	-	9
10	Stool	-	5	-	7
11	Stool	-	8	-	9
12	Blood plasma	-	5	-	-
13	Blood plasma	-	6	-	10
14	Blood plasma	-	3	-	-
15	Blood plasma	-	-	-	10
16	Blood serum	-	-	-	16
17	Blood serum	17	-	-	-

Ct = cycle threshold.

**Table A5:** Comparison of recorded Ct values of the real-time pan-trematode screening PCR assays for samples showing concordant and discordant test results.

	*n*	Mean (±SD)	Median (min., max.)	*p*-Value (Mann–Whitney)
Pan-trematode PCR 1—concordantly positive results in comparison with pan-trematode PCR 2	11	29.1 (± 3.9)	28 (24, 38)	ref.
Pan-trematode PCR 1—discordantly positive results in comparison with pan-trematode PCR 2	4	37.5 (± 2.1)	37.5 (35, 40)	0.0131
Pan-trematode PCR 2—concordantly positive results in comparison with pan-trematode PCR 1	11	29.1 (± 3.9)	28 (24, 38)	ref.
Pan-trematode PCR 2—discordantly positive results in comparison with pan-trematode PCR 1	2	44.0 (± 1.4)	43 (44, 45)	0.0256

Min. = minimum; max. = maximum; ref. = reference; *p*-value = significance level.
